# Electrospun CuO-Nanoparticles-Modified Polycaprolactone @Polypyrrole Fibers: An Application to Sensing Glucose in Saliva

**DOI:** 10.3390/nano8030133

**Published:** 2018-02-27

**Authors:** Ting Xu, Wen Jin, Zhenzhen Wang, Haiyan Cheng, Xinhua Huang, Xiaoyu Guo, Ye Ying, Yiping Wu, Feng Wang, Ying Wen, Haifeng Yang

**Affiliations:** The Education Ministry Key Lab of Resource Chemistry, Shanghai Key Laboratory of Rare Earth Functional Materials, Shanghai Municipal Education Committee Key Laboratory of Molecular Imaging Probes and Sensors and Department of Chemistry, Shanghai Normal University, Shanghai 200234, China; xt_42_2012@163.com (T.X.); Shnujinwen@163.com (W.J.); 13262863105@163.com (Z.W.); cheng7777@163.com (H.C.); 18390183925@139.com (X.H.); gxy2012@shnu.edu.cn (X.G.); yingye@shnu.edu.cn (Y.Y.); yipingwu@shnu.edu.cn (Y.W.); wangfeng@shnu.edu.cn (F.W.)

**Keywords:** electrospinning, polycaprolactone, polypyrrole, CuO NPs, non-enzymatic sensor, glucose in saliva

## Abstract

A non-invasive method for detecting glucose is pursued by millions of diabetic patients to improve their personal management of blood glucose. In this work, a novel CuO nanoparticles (NPs) decorated polycaprolactone@polypyrrole fibers modified indium-tin oxide (denoted as CuO/PCL@PPy/ITO) electrode has been fabricated by electrospinning combined with the electrodeposition method for non-enzymatic detection of glucose in saliva fluid. The electrospun composite fibers exhibit high sensitivity for the glucose detection. The synergistic effect between CuO and PPy together with the unique three-dimensional net structure contributes the reliable selectivity, good test repeatability, large-scale production reproducibility in massive way, the reasonable stability and a high catalytic surface area to the sensor. Quantitative detection of glucose is determined in the linear range from 2 μM to 6 mM and the lowest detection limit is 0.8 μM. The CuO/PCL@PPy/ITO electrode shows potential for the non-invasive detection of salivary glucose.

## 1. Introduction

Diabetes is a chronic and serious disease caused by the metabolic disturbances. It is well known that blood glucose detection is a routine method to monitor the illness’s condition and evaluate the therapeutic effects. However, it brings so much psychological stress and additional pain to the patient [1,2]. It is obvious that the development of responsive and non-invasive methods for detecting glucose is a need for diabetic patients, which could create the benefit of improving personal management of their blood glucose [3].

Saliva is a biological fluid with great important biomarkers for the indication of a person’s health level [4,5,6]. For instance, glucose in saliva, which has good correlation with its concentrations in blood. It is believed that saliva is a promising alternative for non-invasive determination of glucose and might become a better indicator of diseases than blood, due to its easy collection by individuals and with barely any discomfort [7,8,9,10] as compared with other body fluids such as tears and sweat that serve the same purpose.

To date, there have been few articles on the detection of glucose in saliva. This is because the glucose content in saliva is much lower than the detection range of blood glucose in normal sensors. Some studies have tried to use enzymes to improve performance in salivary detection. Lui et al. designed a novel dual-enzyme biosensor composed of glucose oxidase (GOx) and pistol-like DNAzyme (PLDz) to detect glucose levels in tears and saliva [9]. Soni and Jha developed a biosensor for the detection of salivary glucose by using immobilized glucose oxidase enzyme on a filter paper strip [8]. Undoubtedly, the enzyme-based biosensors hold the best selectivity with the highest sensitivity. However, enzyme-based biosensors are limited by their instability of enzyme activity batch-by-batch and their high sensitivity to temperature, pH, and humidity [11,12]. Some other studies have tried to introduce different metal materials to enhance the sensors’ catalytic properties such as silver [13], gold [14], rare metals [15], and alloy [16]. For example, a sensor designed by Du et al. has been built using a layer-by-layer self-assembly to deposit single-walled carbon nanotubes (SWCNTs), chitosan, gold nanoparticles, and graphene onto a screen-printed platinum electrode. The biosensor could be utilized for the quantitative detection of glucose in both buffer solution and saliva samples [7]. An IrO_2_@NiO core-shell nanowire was designed for the detection of low concentration of glucose. The sensor was only capable of distinguish diabetes from healthy people and it doesn’t provide a specific measurement [15]. In a word, most sensors suffer from low sensitivity, poor selectivity and the interference from other species such as chloride, ascorbic acid, uric acid, and dopamine.

Alternatively, copper oxides, have attracted immense attention owing to their excellent electrocatalytic activity to glucose oxidation and relatively low cost [17,18]. As an important p-type metal oxide semiconductor, copper oxide has aroused considerable research interest [19]. Especially, CuO at nanoscale has additional advantages from its high specific surface area, chemical stability, electrochemical activity, and high electron communication features [20]. Nanostructured CuO-based non-enzymatic glucose sensors with good performance have been developed [21,22]. However, the conductivity of CuO as a typical p-type transition metal oxide is quite poor.

Electrospinning is one of the most straightforward and cost-effective methods for producing nanofibers in a large scale way and the resultant fibers present the properties of a large surface area and high porosity [23,24,25,26,27,28,29,30]. In this study, polypyrrole (PPy) was chosen for electrospinning fabrication and textile electrode preparation because PPy is one of the conducting polymers that show favorable thermal stability, high electrical conductivity and ingenious redox properties as well as environmental stability. Polycaprolactone (PCL) as a mixing specie was co-electrospun to enforce the mechanical property the as-produced fibers in addition to its great biocompatibility and slow biodegradability during the electrospinning process [31,32]. The PCL@PPy nanofibers were used as a nanoscale frame to contribute a high specific surface area for the dispersion of the as-made CuO NPs. In this study, the CuO/PCL@PPy fibers were directly collected on the surface of indium-tin oxide (ITO) glass to thus form a non-enzymatic glucose sensor. Under the alkaline conditions of 0.01 M NaOH, the CuO/PCL@PPy/ITO-based sensor with its unique three-dimensional net structure presented reliable selectivity, good sensitivity, and reproducibility and stability to detect glucose in saliva.

## 2. Materials and Methods

### 2.1. Chemicals and Reagent

β-d-(+)-glucose was purchased from Shanghai Macklin Biochemical Co., Ltd. (Shanghai, China) Hydrochloric acid (HCl), sodium hydroxide (NaOH), sodium sulfate (Na_2_SO_4_), copper sulfate pentahydrate (CuSO_4_·5H_2_O), Na_2_HPO_4_, NaH_2_PO_4_, formic acid, acetic acid, dopamine (DA), ascorbic acid (AA), and uric acid (UA) were purchased from Sinopharm Chemical Reagent Co., Ltd. (Shanghai, China) Polycaprolactone, and pyrrole were obtained from Sigma Aldrich (St. Louis, MO, USA). Hexafluoroisopropanol (HFIP) (99.5%) was obtained from Aladdin Industrial Co. (Shanghai, China). Iron (III) chloride anhydrous (98%) and ethanol were provided by Ruijie Chemical Reagent (Shanghai, China). All the chemicals were used as received without any further purification. All solutions were prepared with deionized (DI) water (18 MΩ cm). Phosphate buffer solution (PBS) was prepared with the solution of pH = 7.

### 2.2. Instrument and Equipment

The electrochemical characteristics of ITO, PCL@PPy/ITO, Cu/PCL@PPy/ITO, and CuO/PCL@PPy electrodes were examined by using cyclic voltammetry (CV), amperometric response (i-t), and electrochemical impedance spectra (EIS). Electrochemical experiments were performed with a CHI660C electrochemical workstation (Chen Hua Instruments Co., Shanghai, China). A platinum electrode and a saturated calomel electrode (SCE) were used as auxiliary and reference electrodes, respectively. The morphologies of the modified electrode surface were analyzed by field-emission scanning electron microscopy (FE-SEM, using a JSM-6700F system from JEOL, Tokyo, Japan) and transmission electron microscopy (TEM, JEOL JEM-2000 FX, Tokyo, Japan). The X-ray diffraction (XRD) patterns of CuO/PCL@PPy and PCL@PPy were recorded on a D/Max 2500V/PC X-ray diffractometer (Cu, 40 kV, 200 mA, Rigaku, Tokyo, Japan), at a scan rate of 0.05° s^−1^ with 2 ranging from 10° to 80°.

### 2.3. Preparation of Electrospinning PCL Nanofibers

The PCL nanofibers were prepared by a method similar to previous report [33]. Briefly, the precursor solution consisted of 0.7 g PCL granules, 3 mL HFIP, 1 mL acetic acid (20%) and 1 mL formic acid (60%). The precursor solution was then stirred for 12 h at the room temperature until entirely dissolved. The voltage difference between the stainless steel nozzle (positive) (0.5 mm inner diameter) and the sample collector (negative) (ITO) was about 18 kV. The distance between the stainless steel nozzle and the ITO was about 15 cm. The flow rate of 1 mL/h was applied during the electrospinning.

### 2.4. Preparation of CuO/PCL@PPy/ITO

**Scheme 1:**
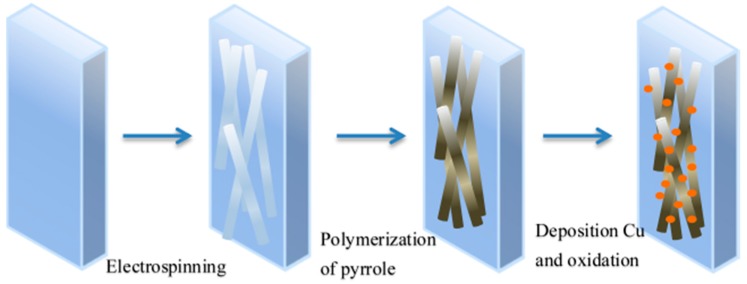
Schematic of fabrication of CuO/PCL@PPy on indium-tin oxide (ITO) electrodes.

The ITO with PCL nanofibers was firstly dipped into a 0.025 mM (15 mL) pyrrole solution containing the solution of 0.04 M FeCl_3_ for 1.5 h until the color of the nanofibers changed from a white color to a light black color. The as-formed PCL@PPy nanofibers were then washed with DI water and dried in the air. The electrochemical deposition of copper onto the substrate of ITO with PCL@PPy nanofibers was then performed via multi-potential steps scan at ambient temperature. Different applied potentials (−0.8, −1.0, 1.2, and −1.4 V versus SCE), different deposition laps (1000, 1200, 1400, and 1600) and various concentrations of copper sulfate (5, 10, 15, and 20 mM) have been investigated for obtaining the optimal electrodeposition condition. The Cyclic Voltammogram (CV) scanning was applied to oxidize the Cu nanoparticles in the solution of 0.1 M NaOH and the potential was scanned between −1 V and 1 V for 10 cycles at the scan rate of 50 mV/s. Scheme 1 describes the above fabrication process.

## 3. Results and Discussion

### 3.1. Characterization of as-Prepared Electrodes

**Figure 1:**
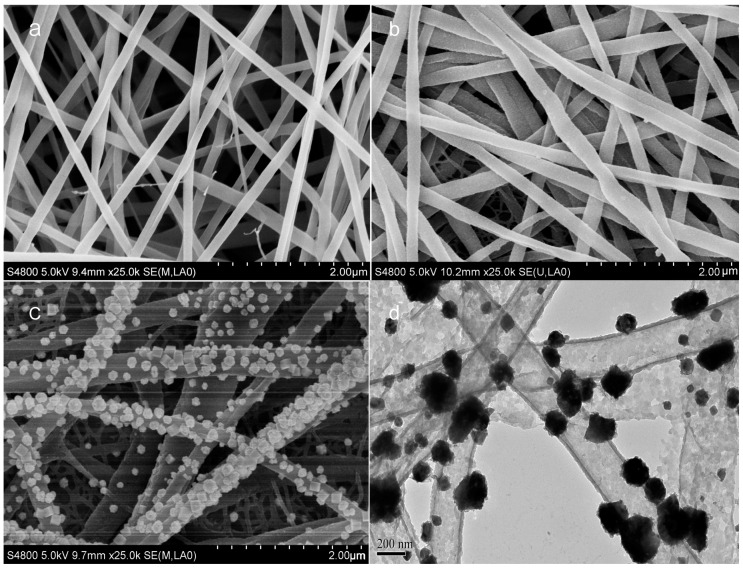
FE-SEM images of (**a**) PCL nanofibers; (**b**) PCL@PPy; (**c**) CuO/PCL@PPy; (**d**) TEM image of CuO/PCL@PPy.

The surface morphology of PPy/PCL and CuO/PPy/PCl were characterized by FE-SEM. As shown in Figure 1a, the PCL nanofibers with diameters varying from 150 to 250 nm are observed to be relatively smooth with no residue attached, which is consistent to with previous work [34]. Figure 1b shows the PPy/PCL nanofibers becoming obviously rough. The modification of PPy led to the formation of core-shell PPy/PCL and the white color of PCL nanofibers turned into the black color of PPy/PCL nanofibers. Figure 1c shows the morphology of CuO/PCL@PPy nanofibers. The CuO NPs are homogeneously and uniformly distributed at the surface of PCL@PPy nanofibers. The average size of the CuO NPs is approximately 100 nm, which may bring the benefits of large surface area, good catalytic activity, and large number of active sites. The TEM image of the CuO/PCL@PPy nanofibers further confirms the well-dispersed state of CuO NPs on the surfaces of the fibers. It also confirms the core-shell structure of the PCL@PPy nanofibers in Figure 1d.

**Figure 2:**
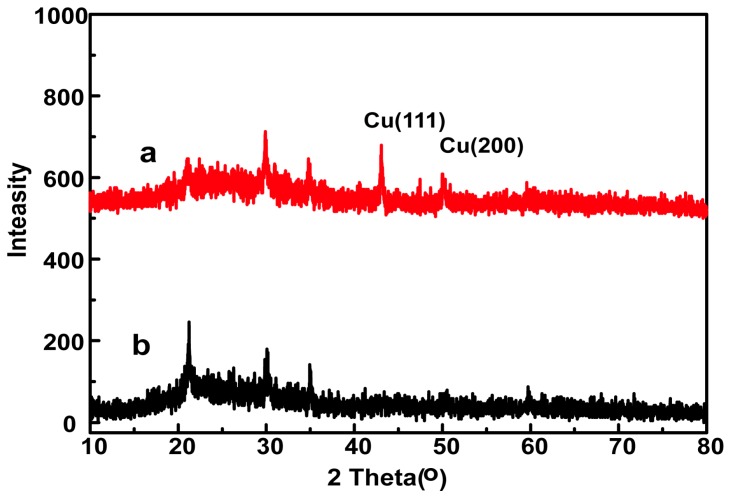
XRD patterns of (a) CuO/PCL@PPy/ITO and (b) PCL@PPy/ITO electrodes.

Figure 2 shows the XRD patterns of the CuO/PPy/PCL and PPy/PCL nanowires. The observed diffraction peaks can be attributed to Cu and the PPy/PCL substrate. The diffraction peaks located at 43.50° and 50.50°, are assigned to the diffraction patterns from the (111) and (200) planes of the cubic lattice of Cu(0) (JCPDS file: 85–1326). No distinct peak for Cu(II) is identified in Figure 2, which might be due to a low crystallinity the CuO NPs.

**Figure 3:**
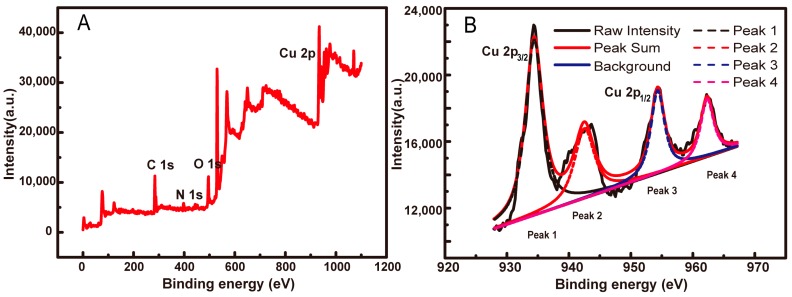
(**A**) XPS survey spectrum and (**B**) deconvoluted Cu 2p spectra for CuO/PCL@PPy/ITO.

In order to clarify the formula of copper oxide, the CuO/PPy/PCL nanofilm was further characterized by XPS. Figure 3A shows the XPS survey spectrum of the CuO/PCL@PPy substrate, which depicts a binding energy peak at 934.4 eV corresponding to the Cu 2p region. In Figure 3B, the strong satellite peaks at 935 and 955 eV confirm the presence of Cu(II) species. The absence of the satellite peak at 939−945 eV suggests the lack of Cu(I). The format of copper within the nanofibers thus includes both Cu(II) oxide and pure Cu(0).

### 3.2. Electrochemical Response of Glucose

**Figure 4:**
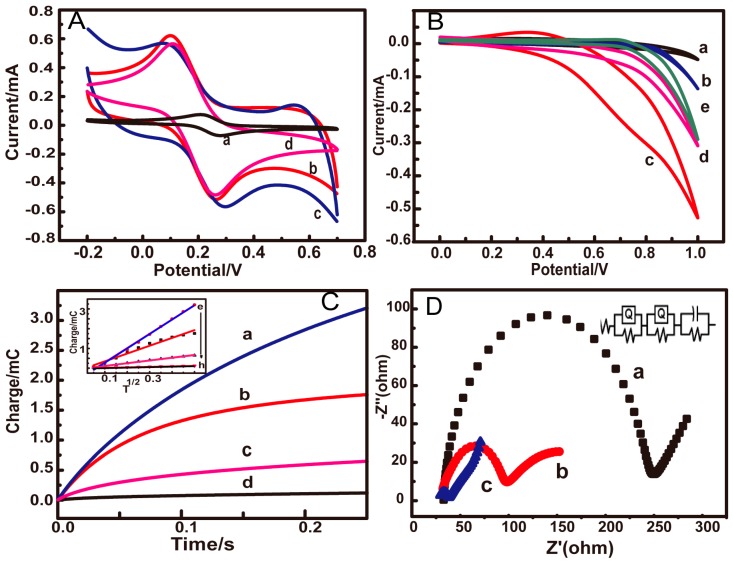
(**A**) The cyclic voltammograms (CVs) of (a) bare ITO; (b) PCL@PPy/ITO; (c) Cu/PCL@PPy/ITO; and (d) CuO/PCL@PPy/ITO in 0.1 M KCl solution containing 0.5 mM K_3_ [Fe(CN)_6_] with the scan rate of 50 mV/s; (**B**) CVs of (a) bare ITO; (b) PCL@PPy/ITO; (c) CuO/PCL@PPy/ITO and (d) Cu/PCL@PPy/ITO electrodes in 0.01 M NaOH solution with 0.1 mM glucose; (e) CuO/PCL@PPy/ITO in 0.01 M NaOH solution without glucose, at a scan rate of 50 mV s^−1^; (**C**) Plot of Q-t curves of (a) Cu/PCL@PPy/ITO; (b) PCL@PPy/ITO; and (c) CuO/PCL@PPy/ITO; (d) bare ITO in 0.1 M KCl soulution containing 0.5 mM K_3_ [Fe(CN)_6_] (Inset of C: plot of Q-t^1/2^ curves on (e) Cu/PCL@PPy/ITO; (f) PCL@PPy/ITO; (g) CuO/PCL@PPy/ITO and (h) bare ITO); (**D**) the Nyqist plots of the EIS of the (a) ITO; (b) CuO/PCL@PPy/ITO; and (c) PCL@PPy/ITO electrodes in 0.1 M KCl solution containing 0.5 mM K_3_ [Fe(CN)_6_].

Figure 4 presents the electrochemical behaviors of bare ITO, PCL@PPy/ITO, Cu/PCL@PPy/ITO and CuO/PCL@PPy/ITO in a solution of 5 mM Fe(CN)_6_^3−^ by using cyclic voltammetry. The anodic peak current of ITO is about 71.51 A, and peak currents of PCL@PPy/ITO and the CuO/PCL@PPy/ITO are 455.6 A and 512.6 A, respectively. Obviously, the large surface area of CuO/PCL@PPy/ITO contributed to the larger electrochemical response. The difference of potentials (Ep = Epa − Epc, where Epa and Epc are the anodic and cathodic peak potentials, respectively) was measured to be 72 mV for ITO, 155 mV for CuO/PCL@PPy/ITO, 266 mV for Cu/PCL@PPy/ITO, and 167 mV for PCL@PPy/ITO. The modification reduced the conductivity and the reversibility of electron transfer as comparison with ITO. It is interesting to note that CuO/PCL@PPy/ITO shows relatively better reversibility than Cu/PCL@PPy/ITO.

The Anson equation was quoted to estimate the effective surface area for electrochemical reaction.

(1) $$Q=\frac{2nFAcD^{1/2}T^{1/2}}{\pi^{1/2}}+Q_{dl}+Q_{ads}$$

where *n* is the number of electron transfers, *F* is Faraday’s constant, *A* is the effective surface area of the working electrode, *c* is the concentration of the reactant, *Q_dl_* is the double layer charge which can be eliminated by background subtraction, and *Q_ads_* is the Faradaic charge. Figure 4C shows the *Q*-*t* curves of various electrodes of CuO/PCL@PPy/ITO, Cu/PCL@PPy/ITO, PCL@PPy/ITO and ITO in the solution containing 0.1 mM K_3_ [Fe(CN)_6_] and 1.0 M KCl. The standard diffusion coefficient (*D*_0_) of K_3_ [Fe(CN)_6_] at 25 °C is 7.6 × 10^−6^ cm^2^∙s^−1^ [35]. The active areas calculated for CuO/PCL@PPy/ITO, Cu/PCL@PPy/ITO, PCL@PPy/ITO and ITO are 0.147, 0.763, 0.416, and 0.026 cm^2^, respectively. Turning Cu into CuO led to a decrease of the active area due to the reduction of the electric conductivity.

The CV curves of the bare ITO, PCL@PPy/ITO, and CuO/PCL@PPy/ITO electrodes in 0.01 M NaOH solution containing 0.1 mM glucose were acquired at the scan rate of 50 mV s^−1^. In Figure 4B, the bare ITO (curve a) and the PCL@PPy-modified ITO (curve b) electrodes show no responses to glucose oxidation. As compared to the CuO/PCL@PPy/ITO electrode without glucose (curve e), the CuO/PCL@PPy/ITO electrode with glucose (curve c) demonstrates a significant current change starting at about +0.50 V and has a shoulder oxidation hump at +0.70 V in the anodic scan. Obviously, the current responded to the addition of glucose. The glucose oxidation at CuO/PCL@PPy/ITO was an irreversible process, suggesting that the CuNPs played a key role in the electrochemical catalytic oxidation.

In the electrode system of CuO/PCL@PPy/ITO, the glucose can be directly oxidized into gluconolactone under the alkaline condition. However, the mechanism of the glucose oxidation at the copper based system remains unclear and might be dependent on the transformation of the Cu redox couples from Cu(II) to Cu(III) [36]. The possible glucose oxidation process at CuO is depicted as follows [37]:

(2) $$\mathrm{CuO}+H_{2}O+{2\mathrm{OH}}^{-}\to {\mathrm{Cu}(\mathrm{OH})}_{4}^{-}+e^{-}$$

(3) $$\mathrm{CuO}+{\mathrm{OH}}^{-}\to \mathrm{CuOOH}+e^{-}(\mathrm{or})$$

(4) Cu(III)+glucose→Gluconolactone+Cu(II)

An EIS experiment has been performed to study the performance of the interface property of the electrode surface. In the presence of the redox probe [Fe(CN)_6_]^4−/3−^, the Nyquist plots of the bare ITO electrode, the PCL@PPy/ITO electrode and the CuO/PCL@PPy/ITO electrode were recorded. In the inset of Figure 4D, by using ZinView software (Scribner Associates, Southern Pines, NC, USA) to fit the impedance spectra (Figure 4D), the equivalent electrical circuits are illustrated. The diameter of a semicircle portion is equal to the electron transfer resistance (*R*_ct_), indicating in the electron-transfer kinetics of the redox reaction at the electrode surface and indirectly reflecting conductivity. The PCL@PPy/ITO electrode exhibits minimum *R*_ct_ and hence the conductivity of PCL@PPy/ITO is the best one.among the electrode types. Clearly, covering electrospun nanofibers covered with PPy improved the conductivity properties with theas a significant increase on thein surface area. Modification was observed. The modification of PCL@PPy/ITO with Cu species containing CuO onto the PCL@PPy/ITO increased *R*_ct_.

### 3.3. Optimization of Preparation Conditions for CuO/PCL@PPy/ITO Electrodes

**Figure 5:**
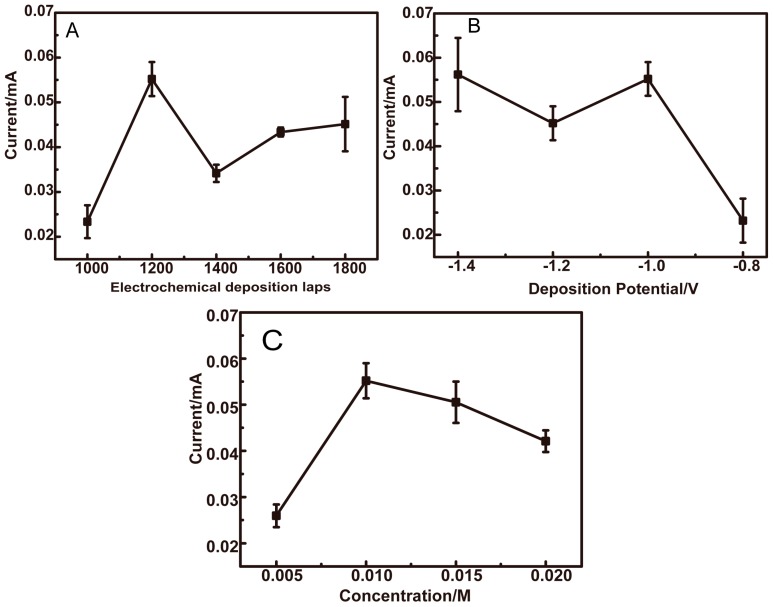
i-t of CuO/PCL@PPy/ITO electrode in 0.01 M NaOH solution containing 0.1 mM glucose (**A**) different electrochemical deposition laps; (**B**) different deposition potentials; (**C**) varying concentrations of CuSO_4_ solution.

The fabrication process of CuO NPs deposition onto the PCL@PPy nanofiber layer has been optimized. The CuO NPs were deposited onto PCL@PPy nanofibers by the electroplating method via multi-potential steps. The concentration of CuSO_4_, the deposition potential, and the electrochemical deposition laps affect the final products of CuO/PCL@PPy, which were investigated by means of i-t measurements in 0.01 M NaOH solution containing 0.01 mM glucose. As seen in Figure 5, the optimal CuO/PCL@PPy with the highest catalysis can be reached when the deposition parameters were set at the usage of 0.01 M CuSO_4_ solution, an applied potential of −1 V and the deposition lap number of 1200 cycles.

**Figure 6:**
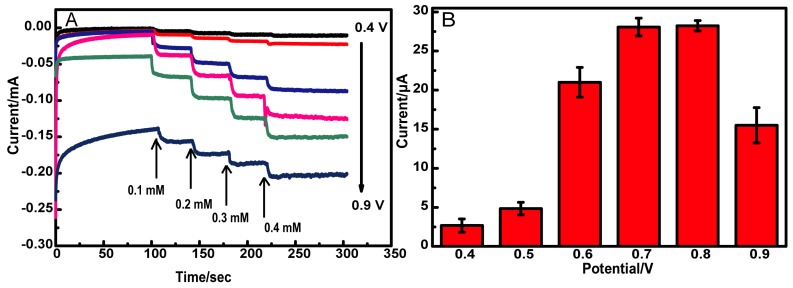
(**A**) i-t curve of the CuO/PCL@PPy/ITO electrode by successively for four times, adding 0.1 mM glucose into 10 mL 0.01 M NaOH solution recorded at different detection potentials: 0.4 V, 0.5 V, 0.6 V, 0.7 V, 0.8 V and 0.9 V; (**B**) Comparable graph for current changes by adding 0.1 mM glucose into 10 mL 0.01 M NaOH solution, recorded at different detection potentials.

Potential-dependent i-t responses were recorded by continuously adding 1 M glucose (1 μL) into 0.01 M NaOH solution under the stirring condition. In Figure 6, the working potential set at 0.7 V shows the relatively better sensitivity than the others, which is selected to conduct the following amperometric determination of glucose.

**Figure 7:**
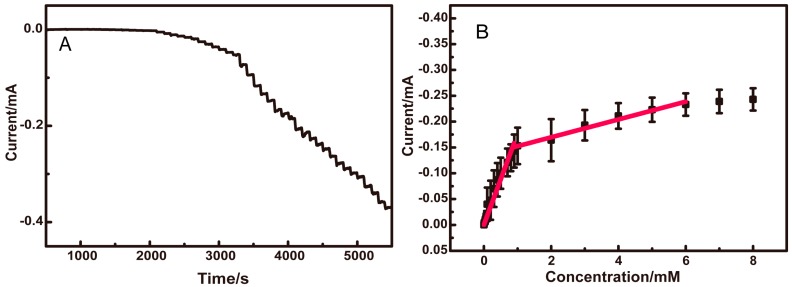
(**A**) i-t curves of CuO/PCL@PPy/ITO response to different concentrations of glucose in 0.01 M NaOH at 0.7 V; (**B**) the corresponding quantitative calibration curves.

Figure 7 exhibits the i-t curve for the detection of glucose by using the CuO/PCL@PPy nanofibers electrode in a stirred solution of 0.01 M NaOH with an optimal potential at +0.70 V. There are two linear relationships between the concentrations of glucose and current responses, ranging from 2 μM to 1 mM and from 1 mM to 6 mM, respectively. It meets the requirement of the determination of glucose in human saliva because of its glucose concentration varying in a range from 2 μM to 6 mM [5,7,8].

### 3.4. Selectivity and Stability Studies

**Figure 8:**
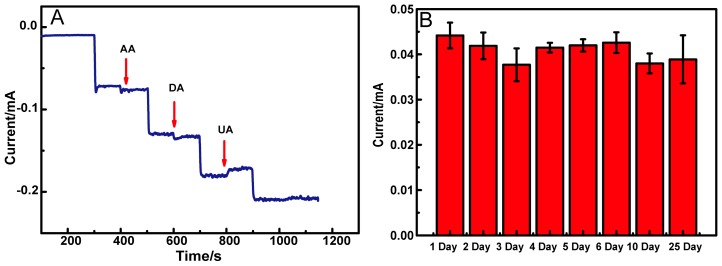
(**A**) Amperometric responses of CuO/PCL@PPy/ITO to successive additions of 1.0 mM glucose and 0.01 mM ascorbic acid (AA), dopamine (DA), and uric acid (UA) in 0.01 M NaOH at −0.7 V; (**B**) The current response of the CuO/PCL@PPy/ITO electrode to 0.1 mM glucose within 25 days.

In order to examine the selectivity for the as-made glucose biosensor, the i-t curves have been taken at 0.7 V in 0.01 M NaOH solution containing 0.1 mM glucose by successively adding the possible interfering species involving 0.01 mM AA, DA, and UA. As seen in Figure 8A, for the CuO/PCL@PPy/ITO electrode, no obvious interference is from the above-observed species, suggesting the good selectivity of the biosensor.

The long-term stability of the CuO/PCL@PPy/ITO electrode was also explored by recording the i-t response toward 0.1 mM glucose over 10 days. As displayed in Figure 8B, the amperometric response can retain approximately 88% of the level of the original value after 25-days of monitoring and storage in ambient conditions. It suggests the reasonable stability of the CuO/PCL@PPy/ITO-based sensor.

### 3.5. Detection of Glucose in Real Samples

As described above, the physiological level of glucose in saliva is in the range of 0.5–20 mg/dL (27.8 μM–1.11 mM). In this work, the saliva sample (1 mL) was centrifuged and the upper side of the liquid was diluted with NaOH solution (0.01 M) up to 10 mL. The spiked samples were obtained by adding the due concentrations of glucose into the treated samples. The results for the detection of glucose in human saliva by using the CuO/PCL@PPy/ITO electrode are summarized in Table 1. The recoveries are in the range from 96.36% to 105.6%. As compared with the other glucose sensors in the literature, as shown in Table 2, the as-prepared CuO/PCL@PPy/ITO electrode demonstrates excellent performance with a wider dynamic concentration range and sufficient sensitivity. The result suggests that the CuO/PCL@PPy/ITO electrode can be developed to be a promising candidate for sensing low concentrations of glucose in samples such as salivary glucose.

## 4. Conclusions

We have successfully constructed a CuO/PCL@PPy/ITO electrode by the electrospinning technique together with the in-situ electrochemical deposition. The resultant CuO/PCL@PPy/ITO with a reasonable stability contributed the excellent electrocatalytic activity for the detection of low concentrations of glucose. The CuO/PCL@PPy/ITO-based glucose sensors can be fabricated in the massive way. The sensor has been successfully utilized to determine the concentration of glucose in human saliva. It is expected that the mass-produced CuO/PCL@PPy/ITO-based glucose sensors made massively can become a non-invasive assay for the detection of salivary glucose at home. 

## Figures and Tables

**Table 1:** Detection of glucose in real samples by using the CuO/PCL@PPy/ITO electrode.

Samples	Added (μM)	Found (μM)	Recovery (%)	RSD (%)
Saliva	0	38.87	0	6.05%
	50	91.47	105.1%	3.26%
	150	197.5	105.6%	5.78%
	250	279.8	96.36%	5.80%

**Table 2:** A comparison of the CuO/PCL@PPy/ITO electrode with other methods.

Modified Electrodes	Detection Limit (μM)	Detection Potential (V)	Linear Range	Reference
Au/ammonia-doped-prGO/CuO	0.25 μM	+0.50	(0.00025–6 mM)	[18]
CuO/rGO	0.1 μM	+0.4 V	0.0004–3.3 mM	[37]
Cu_2_O/Ppy LT paper electrodes	NA	0	1–40 mM	[38]
Ni-Cu/TiO_2_ NTs	5 μM	+0.55 V	0.01–3.2 mM	[39]
CuxO/PPy/Au	6.2 μM	+0.6 V	0–8 mM	[40]
CuxO/Ppy/rGO/GCE	0.03 μM	+0.2 V	0.1–100 mM	[41]
CuO/ITO	0.7 μM	+0.59 V	0–4.4 mM	[42]
NiO/CuO/PANI	2.0 μM	+0.6 V	0.02–2.500 mM	[43]
Cu−Cu_2_O/TiO_2_/Ti electrode	8.6 μM	+0.65 V	0.1–2.5 mM	[44]
NiO/TiO_2_	1.0 μM	+0.47 V	0.005–12.1 mM	[45]
CuO/PCL@PPy/ITO	2 μM	+0.7 V	0.002–6 mM	This work

## References

[1] Newman J.D., Turner A.P. (2005). Home blood glucose biosensors: A commercial perspective. Biosens. Bioelectron..

[2] Wang Z., Lei H., Feng L. (2013). A facile channel for D-glucose detection in aqueous solution. Spectrochim. Acta A Mol. Biomol. Spectrosc..

[3] Nery E.W., Kundys M., Jelen P.S., Jonsson-Niedziolka M. (2016). Electrochemical Glucose Sensing: Is There Still Room for Improvement?. Anal. Chem..

[4] Viswanath B., Choi C.S., Lee K., Kim S. (2017). Recent trends in the development of diagnostic tools for diabetes mellitus using patient saliva. TrAC Trends Anal. Chem..

[5] Malik S., Khadgawat R., Anand S., Gupta S. (2016). Non-invasive detection of fasting blood glucose level via electrochemical measurement of saliva. SpringerPlus.

[6] Elmongy H., Abdel-Rehim M. (2016). Saliva as an alternative specimen to plasma for drug bioanalysis: A review. TrAC Trends Anal. Chem..

[7] Du Y., Zhang W., Wang M.L. (2016). Sensing of Salivary Glucose Using Nano-Structured Biosensors. Biosensors.

[8] Soni A., Jha S.K. (2015). A paper strip based non-invasive glucose biosensor for salivary analysis. Biosens Bioelectron..

[9] Liu C., Sheng Y., Sun Y., Feng J., Wang S., Zhang J., Xu J., Jiang D. (2015). A glucose oxidase-coupled DNAzyme sensor for glucose detection in tears and saliva. Biosens. Bioelectron..

[10] Chen Q., Fu Y., Zhang W., Ye S., Zhang H., Xie F., Gong L., Wei Z., Jin H., Chen J. (2017). Highly sensitive detection of glucose: A quantitative approach employing nanorods assembled plasmonic substrate. Talanta.

[11] Wang G., Lu X., Zhai T., Ling Y., Wang H., Tong Y., Li Y. (2012). Free-standing nickel oxide nanoflake arrays: Synthesis and application for highly sensitive non-enzymatic glucose sensors. Nanoscale.

[12] Zhang Y., Liu Y., Su L., Zhang Z., Huo D., Hou C., Lei Y. (2014). CuO nanowires based sensitive and selective non-enzymatic glucose detection. Sens. Actuators B Chem..

[13] Ngo Y.-L.T., Hoa L.T., Chung J.S., Hur S.H. (2017). Multi-dimensional Ag/NiO/reduced graphene oxide nanostructures for a highly sensitive non-enzymatic glucose sensor. J. Alloys Compd..

[14] Su Y., Luo B., Zhang J.Z. (2016). Controllable Cobalt Oxide/Au Hierarchically Nanostructured Electrode for Nonenzymatic Glucose Sensing. Anal. Chem..

[15] Wang J., Xu L., Lu Y., Sheng K., Liu W., Chen C., Li Y., Dong B., Song H. (2016). Engineered IrO2@NiO Core-Shell Nanowires for Sensitive Non-enzymatic Detection of Trace Glucose in Saliva. Anal Chem..

[16] Yi W., Liu J., Chen H., Gao Y., Li H. (2015). Copper/nickel nanoparticle decorated carbon nanotubes for nonenzymatic glucose biosensor. J. Solid State Electrochem..

[17] Gowthaman N.S.K., Raj M.A., John S.A. (2017). Nitrogen-Doped Graphene as a Robust Scaffold for the Homogeneous Deposition of Copper Nanostructures: A Nonenzymatic Disposable Glucose Sensor. ACS Sustain. Chem. Eng..

[18] Maaoui H., Singh S.K., Teodorescu F., Coffinier Y., Barras A., Chtourou R., Kurungot S., Szunerits S., Boukherroub R. (2017). Copper oxide supported on three-dimensional ammonia-doped porous reduced graphene oxide prepared through electrophoretic deposition for non-enzymatic glucose sensing. Electrochim. Acta.

[19] Gawande M.B., Goswami A., Felpin F.X., Asefa T., Huang X., Silva R., Zou X., Zboril R., Varma R.S. (2016). Cu and Cu-Based Nanoparticles: Synthesis and Applications in Catalysis. Chem. Rev..

[20] Jia W., Guo M., Zheng Z., Yu T., Wang Y., Rodriguez E.G., Lei Y. (2008). Vertically Aligned CuO Nanowires Based Electrode for Amperometric Detection of Hydrogen Peroxide. Electroanalysis.

[21] Zhang X., Gu A., Wang G., Wei Y., Wang W., Wu H., Fang B. (2010). Fabrication of CuO nanowalls on Cu substrate for a high performance enzyme-free glucose sensor. CrystEngComm.

[22] Zhuang Z., Su X., Yuan H., Sun Q., Xiao D., Choi M.M. (2008). An improved sensitivity non-enzymatic glucose sensor based on a CuO nanowire modified Cu electrode. Analyst.

[23] Li L., Wang X., Liu G., Wang Z., Wang F., Guo X., Wen Y., Yang H. (2015). Reproducible preparation of a stable polypyrrole-coated-silver nanoparticles decorated polypyrrole-coated-polycaprolactone-nanofiber-based cloth electrode for electrochemical sensor application. Nanotechnology.

[24] Yoshimoto H., Shin Y.M., Terai H., Vacanti J.P. (2003). A biodegradable nanofiber scaffold by electrospinning and its potential for bone tissue engineering. Biomaterials.

[25] Bhardwaj N., Kundu S.C. (2010). Electrospinning: A fascinating fiber fabrication technique. Biotechnol. Adv..

[26] Zhang B., Zhang Z.G., Yan X., Wang X.X., Zhao H., Guo J., Feng J.Y., Long Y.Z. (2017). Chitosan nanostructures by in situ electrospinning for high-efficiency PM 2.5 capture. Nanoscale.

[27] Sridhar R., Lakshminarayanan R., Madhaiyan K., Barathi V.A., Lim K.H., Ramakrishna S. (2015). Electrosprayed nanoparticles and electrospun nanofibers based on natural materials: Applications in tissue regeneration, drug delivery and pharmaceuticals. Chem. Soc. Rev..

[28] Sha M., Zhang H., Nie Y., Nie K., Lv X., Sun N., Xie X., Ma Y., Sun X. (2017). Sn nanoparticles@nitrogen-doped carbon nanofiber composites as high-performance anodes for sodium-ion batteries. J. Mater. Chem. A.

[29] Yadav K., Nelson C.T., Hsu S.L., Hong Z., Clarkson J.D., Schleputz C.M., Damodaran A.R., Shafer P., Arenholz E., Dedon L.R. (2016). Observation of polar vortices in oxide superlattices. Nature.

[30] Hanson R., Kouwenhoven L.P., Petta J.R., Tarucha S., Vandersypen L.M.K. (2007). Spins in few-electron quantum dots. Rev. Mod. Phys..

[31] Du Y., Chen X., Koh Y.H., Lei B. (2014). Facilely fabricating PCL nanofibrous scaffolds with hierarchical pore structure for tissue engineering. Mater. Lett..

[32] Wei G., Li C., Fu Q., Xu Y., Li H. (2015). Preparation of PCL/silk fibroin/collagen electrospun fiber for urethral reconstruction. Int. Urol. Nephrol..

[33] Wang Z., Ying Y., Li L., Xu T., Wu Y., Guo X., Wang F., Shen H., Wen Y., Yang H. (2017). Stretched graphene tented by polycaprolactone and polypyrrole net–bracket for neurotransmitter detection. Appl. Surf. Sci..

[34] Saeed K., Park S.-Y., Lee H.-J., Baek J.-B., Huh W.-S. (2006). Preparation of electrospun nanofibers of carbon nanotube/polycaprolactone nanocomposite. Polymer.

[35] Li Y., Gao Y., Cao Y., Li H. (2012). Electrochemical sensor for bisphenol A determination based on MWCNT/melamine complex modified GCE. Sens. Actuators B Chem..

[36] Kang X., Mai Z., Zou X., Cai P., Mo J. (2007). A sensitive nonenzymatic glucose sensor in alkaline media with a copper nanocluster/multiwall carbon nanotube-modified glassy carbon electrode. Anal. Biochem..

[37] Wang X., Liu E., Zhang X. (2014). Non-enzymatic glucose biosensor based on copper oxide-reduced graphene oxide nanocomposites synthesized from water-isopropanol solution. Electrochim. Acta.

[38] Periasamy P., Roy P., Wu W.-P., Huang Y.-H., Chang H.-T. (2016). Glucose Oxidase and Horseradish Peroxidase Like Activities of Cuprous Oxide/Polypyrrole Composites. Electrochim. Acta.

[39] Li X., Yao J., Liu F., He H., Zhou M., Mao N., Xiao P., Zhang Y. (2013). Nickel/Copper nanoparticles modified TiO_2_ nanotubes for non-enzymatic glucose biosensors. Sens. Actuators B Chem..

[40] Meng F., Shi W., Sun Y., Zhu X., Wu G., Ruan C., Liu X., Ge D. (2013). Nonenzymatic biosensor based on Cu(x)O nanoparticles deposited on polypyrrole nanowires for improving detection range. Biosens. Bioelectron..

[41] Nia P.M., Meng W.P., Lorestani F., Mahmoudian M.R., Alias Y. (2015). Electrodeposition of copper oxide/polypyrrole/reduced graphene oxide as a nonenzymatic glucose biosensor. Sens. Actuators B Chem..

[42] Wang T., Su W., Fu Y., Hu J. (2016). Controllably annealed CuO-nanoparticle modified ITO electrodes: Characterisation and electrochemical studies. Appl. Surf. Sci..

[43] Ghanbari K., Babaei Z. (2016). Fabrication and characterization of non-enzymatic glucose sensor based on ternary NiO/CuO/polyaniline nanocomposite. Anal. Biochem..

[44] Yang Q., Long M., Tan L., Zhang Y., Ouyang J., Liu P., Tang A. (2015). Helical TiO_2_ Nanotube Arrays Modified by Cu-Cu_2_O with Ultrahigh Sensitivity for the Nonenzymatic Electro-oxidation of Glucose. ACS Appl. Mater. Interfaces.

[45] Gao Z.D., Han Y., Wang Y., Xu J., Song Y.Y. (2013). One-Step to Prepare Self-Organized Nanoporous NiO/TiO_2_ Layers and Its Use in Non-Enzymatic Glucose Sensing. Sci. Rep..

